# Aqueous extract of *Swietenia macrophylla* leaf exerts an anti-inflammatory effect in a murine model of Parkinson’s disease induced by 6-OHDA

**DOI:** 10.3389/fnins.2024.1351718

**Published:** 2024-02-21

**Authors:** Váldina Solimar Lopes Cardoso, Anderson Valente-Amaral, Rayan Fidel Martins Monteiro, Clarina Loius Silva Meira, Natália Silva de Meira, Milton Nascimento da Silva, João de Jesus Viana Pinheiro, Gilmara de Nazareth Tavares Bastos, João Soares Felício, Elizabeth Sumi Yamada

**Affiliations:** ^1^Experimental Neuropathology Laboratory, João de Barros Barreto University Hospital, Federal University of Pará, Belém, Brazil; ^2^Oncology Research Center and Graduate Program in Oncology and Medical Sciences, João de Barros Barreto University Hospital, Federal University of Pará, Belém, Brazil; ^3^Graduate Program in Neuroscience and Cellular Biology, Institute of Biological Sciences, Federal University of Pará, Belém, Brazil; ^4^Neuroinflammation Laboratory, Institute of Biological Sciences, Federal University of Pará, Belém, Brazil; ^5^Liquid Chromatography Laboratory, Institute of Exact and Natural Science, Federal University of Pará, Belém, Brazil; ^6^Laboratory of Pathological Anatomy and Immunohistochemistry, School of Dentistry, Federal University of Pará, Belém, Brazil; ^7^Endocrinology Division, University Hospital João de Barros Barreto, Federal University of Pará, Belém, Brazil

**Keywords:** *Swietenia macrophylla*, natural products, polyphenols, Parkinson’s disease, neuroinflammation, 6-OHDA model, nigrostriatal pathway

## Abstract

**Introduction:**

Parkinson’s disease affects 2% of the population aged over 65 years and is the second most common neurodegenerative disorder in the general population. The appearance of motor symptoms is associated with the degeneration of dopaminergic neurons in the nigrostriatal pathway. Clinically significant nonmotor symptoms are also important for severe disability with disease progression. Pharmacological treatment with levodopa, which involves dopamine restitution, results in a temporary improvement in motor symptoms. Among the mechanisms underlying the pathogenesis of the disease are exacerbated oxidative stress, mitochondrial dysfunction, and neuroinflammation. A phytochemical prospecting study showed that the aqueous extract of the leaves from *Swietenia macrophylla* (Melineaceae), known as mahogany, has polyphenols with antioxidant and anti-inflammatory capacity in a significantly higher percentage than leaf extracts from other Amazonian plants. Furthermore, the antioxidant and anti-inflammatory capacity of aqueous extract of mahogany leaf has already been demonstrated in an *in vitro* model. In this study, we hypothesized that the aqueous extract of mahogany leaf (AEML) has a neuroprotective effect in a murine model of Parkinson’s disease induced by 6-hydroxidopamine (6-OHDA), due to antioxidant and anti-inflammatory properties of its phenolic compounds.

**Methods:**

Mice were treated daily with the mahogany extract at a dose of 50 mg/kg, starting 7 days before 6-OHDA infusion until post-surgery day 7.

**Results and discussion:**

The animals from the 6-OHDA/mahogany group, which corresponds to animals injected with the toxin and treated with aqueous extract of the mahogany leaf, presented distinct behavioral phenotypes after apomorphine challenge and were therefore subdivided into 2 groups, 6-OHDA/mahogany F1 and 6-OHDA/mahogany F2. The F1 group showed a significant increase in contralateral rotations, whereas the F2 group did not show rotations after the apomorphine stimulus. In the F1 group, there was an increase, although not significant, in motor performance in the open field and elevated plus maze tests, whereas in the F2 group, there was significant improvement, which may be related to the lesser degree of injury to the nigrostriatal dopaminergic pathway. The TH+ histopathological analysis, a dopaminergic neuron marker, confirmed that the lesion to the nigrostriatal dopaminergic pathway was more pronounced in 6-OHDA/mahogany F1 than in 6-OHDA/mahogany F2. Our main result consisted of signs of improvement in the inflammatory profile in both the F1 and F2 6-OHDA/mahogany groups, such as a lower number of IBA-1+ microglial cells in the ventral striatum and substantia nigra pars compacta and a reduction in GFAP+ expression, an astrocyte marker, in the dorsal striatum. In this study, several bioactive compounds in the aqueous extract of mahogany leaf may have contributed to the observed beneficial effects. Further studies are necessary to better characterize their applicability for treating chronic degenerative diseases with inflammatory and oxidative bases, such as Parkinson’s disease.

## Introduction

Parkinson’s disease (PD) is the second most common neurological disorder with a complex evolution, affecting 2% of the population over the age of 65 and 4% of the population over the age of 80 (Pringsheim et al., 2014; Llibre-Guerra et al., 2022; Okunoye et al., 2022). Currently, the clinical diagnosis of PD is made based on cardinal symptoms: bradykinesia associated with resting tremor, muscle rigidity, and/or postural instability (Kobylecki, 2020; Tolosa et al., 2021). In addition, significant non-motor symptoms, such as anxiety, olfactory disorders, depression, sleep disorders, and autonomic nervous system impairments, are frequently observed. In some patients, these non-motor alterations precede classic motor disorders; in others, they arise with disease progression and contribute to severe disability and reduced quality of life (Braak et al., 2004; Chaudhuri et al., 2006; Tibar et al., 2018).

Although the pathology of PD involves several areas of the basal ganglia, the degeneration of dopaminergic neurons of the nigrostriatal pathway is associated with the onset and evolution of motor symptoms (Kalia and Lang, 2015; Balestrino and Schapira, 2020; MacMahon Copas et al., 2021) However, the cause of PD remains unknown.

Currently, no therapeutic intervention can successfully delay or stop the progression of PD. Therefore, there are not disease-modifying treatments available. The identification of pathophysiological mechanisms underlying PD creates opportunities for the appropriate development of new therapies with disease-modifying potential based on natural agents or compounds that consistently block the primary mechanisms involved in neuronal death (Del Rey et al., 2018; Hung and Schwarzschild, 2020; Jankovic and Tan, 2020).

*Swietenia macrophylla* is a large tree, known as mahogany, that naturally occurs in tropical and subtropical countries and has ethnomedicinal importance in India, Malaysia, China, and Central and South America (Moghadamtousi et al., 2013). In these countries, traditional people use different parts of this plant for antimicrobial, antioxidant, and antidiabetic purposes. Phytochemical prospecting and biological activity studies found 9 phenolic acids and 18 flavonoids in the aqueous extract from the leaves of *Swietenia macrophylla*, and have demonstrated its antioxidant and anti-inflammatory activity in an *in vitro* model (Pamplona et al., 2015).

Furthermore, the percentage of polyphenols in the aqueous extract of *Swietenia macrophylla* leaf is significantly higher than that in leaf extracts from other Amazonian plants obtained by the same method (Silva et al., 2007). Polyphenols are naturally occurring, non-enzymatic antioxidant compounds that, in low concentrations, are capable of counteracting exacerbated oxidative stress (Kujawska and Jodynis-Liebert, 2018; Ciulla et al., 2019; Singh et al., 2020) In this study, we hypothesized that the aqueous extract of the *Swietenia macrophylla* leaf has neuroprotective effects in a murine model of PD.

## Materials and methods

### Preparation of the plant extract

The aqueous extract of the mahogany leaf (*Swietenia macrophylla* King) was provided by Dr. Milton da Silva from the Chemistry Institute of the Federal University of Pará. The aerial parts of the plant were identified by a botanist from the Botany Department of the Federal Rural University of Pará, Amazonia, Belém, Pará, Brazil, and a specimen of N° 1,320 was deposited in the Institution’s herbarium. The final dilution of the extract was made in physiological saline solution. For more details, see Pamplona et al., 2015.

### Animals, treatment, and stereotaxic intervention

We used 28 Swiss male adult mice, weighing between 40–45 g, eight weeks old, from the Evandro Chagas Institute, Ananindeua, Pará; the mice were randomly separated and kept five animals per cage at a controlled temperature of 23 ± 1° C, light/dark cycle of 12/12 h, with water and balanced feed for rodents *ad libitum.* The time for acclimatization of the animals in the experimental laboratory was five days. All procedures were approved by the Ethics Committee on the Use of Animals of the Federal University of Pará under Technical Report No. 4046270619.

The safety of the aqueous extract of the mahogany leaf (AEML) was previously defined in a subacute oral toxicity trial (data not shown). Based on this result and aiming at allometric conversion, which estimates the use of the equivalent dose as a treatment strategy in humans, we used a dose of 50 mg/kg/day of extract, orally (gavage), for treating animals injected with 6-OHDA or vehicle.

Initially, mice were randomly divided into two groups, one group being pre-treated with aqueous extract of the Mahogany leaf (AEML; *n* = 16) and the other group receiving vehicle (*n* = 12), both orally (gavage), daily, in the morning, for 14 consecutive days. After eight days of treatment, the animals underwent stereotaxis surgery for intrastriatal infusion of 10 μg of 6-OHDA-HCl (Sigma-Aldrich©/A4544) diluted in 2 μL of ascorbic acid (Sigma-Aldrich, USA) or infusion of 2 μL of 0.2% ascorbic acid.

**Figure 1:**
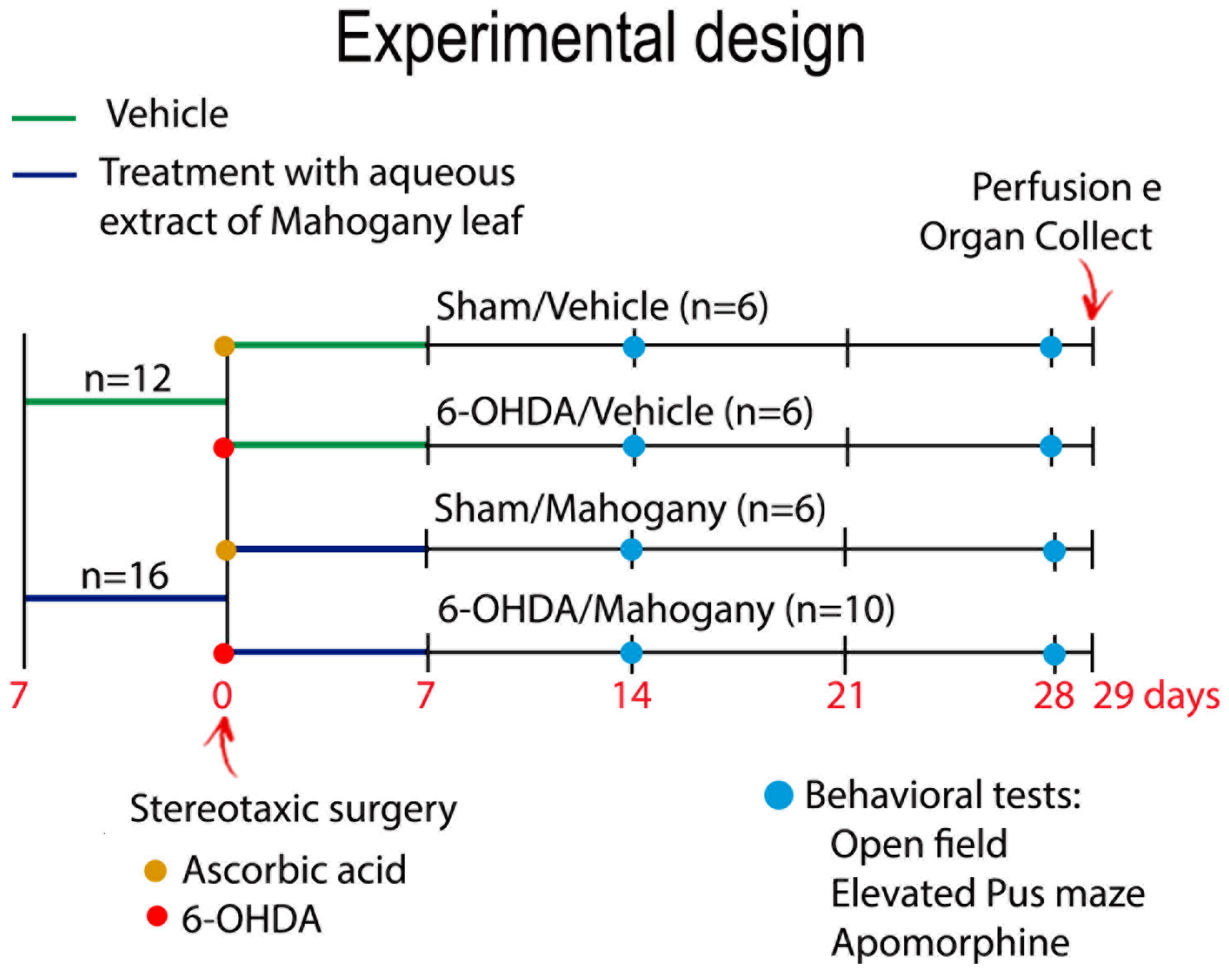
Experimental design. Animals were treated with aqueous extract of the mahogany leaf (AEML) at a dose of 50 mg/kg (*n* = 16) or 0.9% NaCl (*n* = 12) for 14 consecutive days (p.o). On the 8th day of the experiment, the animals underwent stereotaxic surgery for intrastriatal infusion of 10 μg of 6-OHDA (yellow circle) or ascorbic acid (red circle). After stereotaxic surgery, the experimental groups were renamed as follows: sham/vehicle, received ascorbic acid + saline solution (*n* = 6); sham/mahogany, received ascorbic acid + AEML (*n* = 6); 6-OHDA/vehicle, received 6-OHDA + saline solution (*n* = 6); and 6-OHDA/mahogany, received 6-OHDA + AEML (*n* = 10). On the 21st and 35th days of the experiment, open field, elevated plus maze, and apomorphine tests were performed (blue circle). Euthanasia was performed on the 36th day.

The experimental groups were then renamed as follows: sham/vehicle, received ascorbic acid + saline solution (*n* = 6); sham/mahogany, received ascorbic acid + AEML (*n* = 6); 6-OHDA/vehicle, received 6-OHDA + saline solution (*n* = 6); and 6-OHDA/mahogany, received 6-OHDA + AEML (*n* = 10) (Figure 1).

The experimental design of pre-treatment and post-surgery treatment with AEML aimed to achieve a positive pharmacological effect, preventing damage to the nigrostriatal dopaminergic pathway, caused by the infusion of the toxin. A pilot study of 6-OHDA injection into the dorsal striatum was conducted, and based on statistical analysis, the number of animals used in this study was determined.

Before stereotaxic surgery, the animals were anesthetized with a combination of 75 mg/kg ketamine chloride and 10 mg/kg xylazine hydrochloride ip. They were then positioned in the stereotaxic apparatus (Insight Ltda/EFF333) following the coordinates AP +0.8 mm from the bregma, ML +1.5 mm from the sagittal suture, and DV 3.2 mm from the dura mater (Paxinos and Franklin, 2004). A Hamilton syringe was used to inject 2 μL of 6-OHDA or vehicle into the left dorsal striatum. The injection cannula was kept at the infusion site for 5 min before being slowly withdrawn.

### Behavioral tests

All animals were evaluated in the apomorphine-induced rotation test, open field (OF), and elevated plus maze (EPM) on the 14th and 28th day after surgery. In the behavioral assessment room, luminosity was 25–26 Lux (Instrutemp©) and room temperature was 23° ± 1° C; the animals had 1 h of acclimatization and 3 h interval between behavioral assessments. After each evaluation, the apparatus was cleaned with 10% ethanol.

#### Apomorphine (APO)-induced rotation test

Rotational behavior was induced by apomorphine (Sigma-Aldrich/A4393) at a dose of 0.1 mg/kg (sc), and the animals were evaluated for 20 min in a 30 cm diameter arena. The number of rotations is expressed as absolute values.

#### Open field test (OF)

The open field test was used to evaluate spontaneous locomotion and exploratory activity. The animals were placed in the center of a circular acrylic box with gray walls, measuring 30 cm in height and 30 cm in diameter, for free exploration for 5 min. The videos were analyzed using *ANY-maze USA Software, a video tracking system, version 6.12* (Stoelting Co©, USA), in which the perimeter of the central and peripheral zones was defined. The following behavioral parameters were evaluated: total distance traveled (cm), average speed (cm/s), time and distance traveled in the periphery, and time and distance traveled in the center.

#### Elevated plus maze (EPM)

The elevated plus maze was used to measure general activity and anxiety-like behavior. The apparatus was 30 cm long and 5 cm wide, with two open and opposing arms, with side edges 0.5 cm high, to ensure that no animal fell from the apparatus, and two closed arms with walls 15 cm high and opposite. The four arms were connected by a 5 cm^2^ central platform, and the entire structure was elevated 45 cm from the ground. Each trial lasted 5 min and involved placing the mouse on the central platform with its head facing one of the open arms.

Entry into the arms was considered when all paws crossed the line between the central platform and the arm. Using *the ANY-maze video tracking system, version 6.12* (Stoelting Co©, USA), we outlined the perimeter of the open and closed arms and the central zone. The following parameters were analyzed: total distance traveled (cm), total number of entries into open and closed arms (EOCA), percentage of time spent in open arms (tOA), percentage of time spent in closed arms (tCA), number of entries into open arms (EOA), and number of entries into closed arms (ECA).

### Euthanasia and immunohistochemistry

The animals were euthanized with 150 mg/kg of ketamine chloride and 30 mg/kg of xylazine hydrochloride ip; then, transcardial perfusion with 0.1 M PBS and fixation with 4% paraformaldehyde (PFA) were initiated. The brains were collected and post-fixed in 4% PFA for 48 h and then kept in a 30% sucrose solution for 72 h, at 6°C. Subsequently, using a CM1850 cryostat (*Leica Biosystems, Nussloch, Germany*), 40 μm-thick coronal sections of the brain were collected and alternately distributed in six series. After this step, the sections were incubated in the following solutions containing 0.1 M PBS: 0.3% H_2_O_2_ for 30 min to inactivate endogenous peroxidase activity; 0.3% Triton X-100 for 10 min to permeabilize the sections; and normal donkey serum (NDS; Milipore©) at 5% for 1 h to block nonspecific binding.

After this step, each set of sections was incubated in specific primary antibodies, namely: (i) anti-TH (anti-tyrosine hydroxylase; 1:5.000; *Millipore©, polyclonal, anti-rabbit, AB152*); (ii) anti-IBA-1 (anti-ionized calcium binding adapter molecule 1; 1:10.000, *Wako Pure Chemical Industries Ltd, polyclonal, anti-rabbit #019-19741*); and (iii) anti-GFAP (anti-glial fibrilar acidic protein; 1:10.000; *Millipore©, monoclonal, anti-mouse, G3893*), all for 72 h, at 6°C.

The sections were washed three times for 10 min and incubated in their respective secondary antibodies conjugated to biotin, as follows: donkey anti-rabbit (1:500; *Jackson ImmunoResearch©, 711-065-152*); and donkey anti-mouse (1:500; *Jackson ImmunoResearch©, 711-065-150*), for two 12 h at 6°C. The sections were incubated in the avidin–biotin complex (1:200; *Vectastain Elite®, ABC kit; Vector Laboratories©*) for 1 h.

In the final step, we used DAB-Ni as a substrate to visualize the complex formed from the anti-TH and anti-IBA-1 primary antibodies; and 1% 3,3′-diaminobenzidine solution (DAB; Sigma-Aldrich©, D12384) to visualize the complex formed from the anti-GFAP primary antibody. The sections were washed again, mounted, and dehydrated, and only the series immunostained for GFAP were counterstained with cresyl violet.

### Optical density (OD)

TH+ and GFAP+ immunostaining in the dorsal and ventral striatum was analyzed by optical density (OD). We measured three representative sections from the following AP positions: +0.74, +0.38, and + 0.02 mm from the bregma, according to The Mouse Stereotaxy Atlas (Paxinos and Franklin, 2004). The images were captured using a digital camera (*Leica Microsystem Ltd., DFC450, Germany*) attached to a stereomicroscope (*Leica Microsystem Ltd., M205A, Germany*) with image acquisition software (*Leica Application Suit, version 4.2.0, Switzerland*).

The images were transformed into grayscale (8-bit), and optical density quantification was performed using *Fiji ImageJ software* (*version 1.52, National Institutes of Health, USA*). The boundary between the dorsal and ventral STR was defined by a manually drawn horizontal line. In the first two sections, the anatomical reference was the most dorsal point of the lateral ventricle, and in the third section, the anterior commissure, which was adapted from Heuer et al. (2012). Data are expressed as percentages of the contralateral side.

### Stereology

The number of microglia in the ventral and dorsal striatum was determined using stereology. From each animal, we used three representative sections from the following anterior–posterior positions: +0.74, +0.38, and, +0.02 mm from the bregma. We defined the counting limit between the dorsal and ventral striatum as the same as that used for the optical density analysis. In the SNpc, the estimation of the number of neurons and glia was also obtained by stereology; we used six representative coronal sections of the midbrain, located between anterior–posterior coordinates-2.80 mm to-3.80 mm from the bregma, according to The Mouse Stereotaxy Atlas (Paxinos and Franklin, 2004).

For the delimitation of the striatum and SNpc, we used 2x magnification, and cells were counted at 40x magnification. We used the STEREOLOGER software (*Stereology Resource Center, Version Inc., 11.0*) coupled to a Nikon Labophot-2 microscope with a control platform for movement of the X, Y, and Z coordinates (*Prior ES111US, Prior Scientific, Fulbourn, Cambridge*). The results were expressed as a percentage in relation to the contralateral side, and the error coefficient was defined as ≤0.1 (Gundersen et al., 1999).

### Statistical analysis

The results for apomorphine are expressed as absolute values and were analyzed using a two-tailed paired t-test. Open field and elevated plus maze results are expressed as mean ± SEM. In the Open Field, analysis we applied *two-way ANOVA* and for the other comparisons *one-way* ANOVA and Tukey post-test. In the elevated plus maze, we applied *one-way* ANOVA and Tukey *post hoc* test.

Optical density and stereology data are expressed as mean ± SEM and in statistical analysis, we used *one-way ANOVA*, followed by Tukey *post hoc* test. In all analyses, statistical significance was defined as 95% reliability (*p* < 0.05). We used *GraphPad Prism Inc. software (Version 6.01, 2012)* for statistical analyses and graph creation.

## Results

### Behavioral tests

#### Assessment of rotations induced by apomorphine

**Figure 2:**
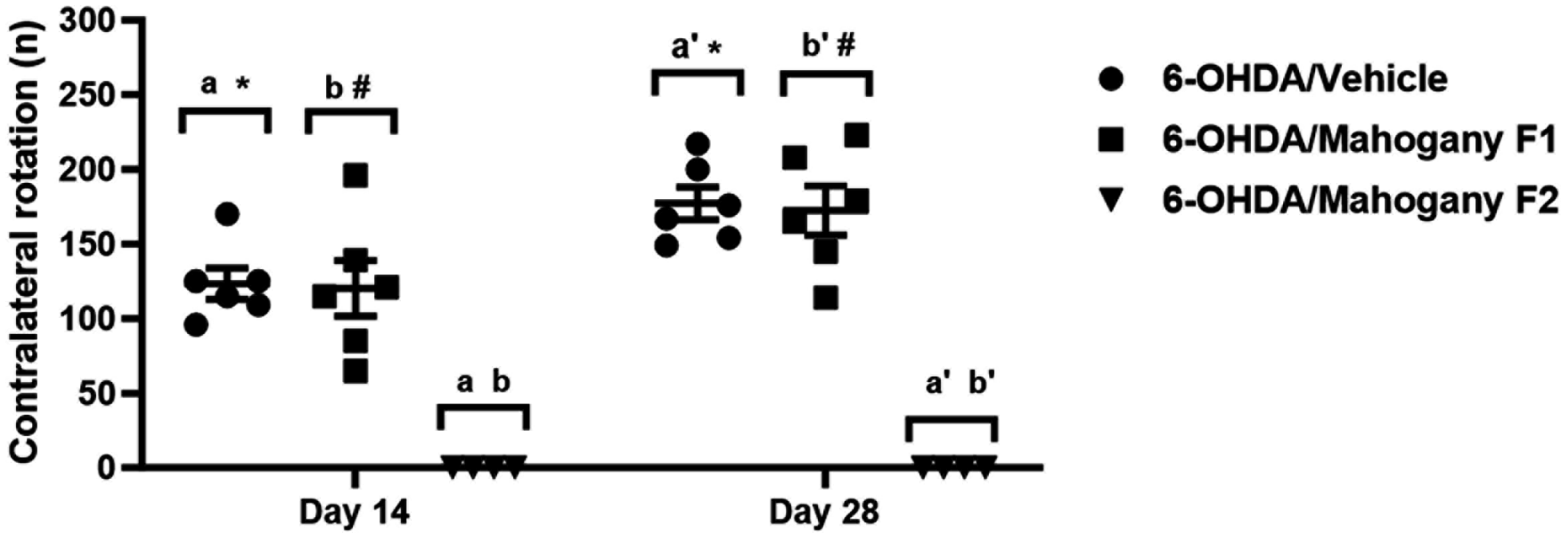
Evaluation of contralateral rotations stimulated by apomorphine in animals injected with 6-OHDA and treated with aqueous extract of the mahogany leaf. The symbols indicate: ^a^6-OHDA/vehicle vs. 6-OHDA/mahogany F2, day 14, *p* < 0.0001. ^b^6-OHDA/mahogany F1vs 6-OHDA/mahogany F2, day 14, *p* < 0.0001. ^a’^6-OHDA/vehicle vs. 6-OHDA/mahogany F2, day 28, *p* < 0.0001. ^b’^6-OHDA/ mahogany F1 vs. 6-OHDA/mahogany F2, day 28, *p* < 0.0001. *6-OHDA/vehicle, day 14 vs. day 28; *p* = 0.0102. ^#^6-OHDA/mahogany F1, day 14 vs. day 28; *p* = 0.0126. The results are expressed in mean ± SEM. Two-way ANOVA followed by Tukey’s *post hoc* test was used, and statistical significance was defined with 95% confidence (*p* < 0.05).

Contralateral rotations induced by apomorphine were evaluated on the 14th and 28th day after surgery. As expected, animals in the sham/vehicle (*n* = 6) and sham/mahogany (*n* = 6) groups did not rotate after the apomorphine challenge. In the 6-OHDA/vehicle (*n* = 6) group, the average number of rotations on the 14th day post-surgery was 123.0 ± 10.3, with a rate of 6.2 ± 0.5 rotations/min; on the 28th day, the average rotations increased significantly, reaching 177.2 ± 10.9 and a rate of 8.9 ± 0.5 rotations/min (*p* = 0.0102) (Figure 2).

In the 6-OHDA/mahogany group (*n* = 10), 40% of the animals did not present contralateral rotations in the apomorphine challenge. Therefore, we subdivided this group according to the presence or absence of contralateral turns. Animals with contraversive behavior were included in the 6-OHDA/mahogany F1 group (*n* = 6), and animals that did not present contralateral rotations were included in the 6-OHDA/mahogany F2 group (*n* = 4).

In the first apomorphine test, the 6-OHDA/mahogany F1 group presented contralateral turns of 120.2 ± 18.6 (mean ± SEM) at a rate of 6.0 ± 0.9 rotations/min. In the second test, the average number of rotations increased to 172.3 ± 16.4 with a rate of 8.6 ± 0.8 rotations/min (*p* = 0.0126). These results indicate that the nigrostriatal dopaminergic lesion in the 6-OHDA/mahogany F1 group was progressive, and treatment with the aqueous extract of the mahogany leaf did not modify the standard motor behavior in the apomorphine test (Figure 2).

#### Assessment of locomotor activity and anxiety-like behavior in the open field test

**Figure 3:**
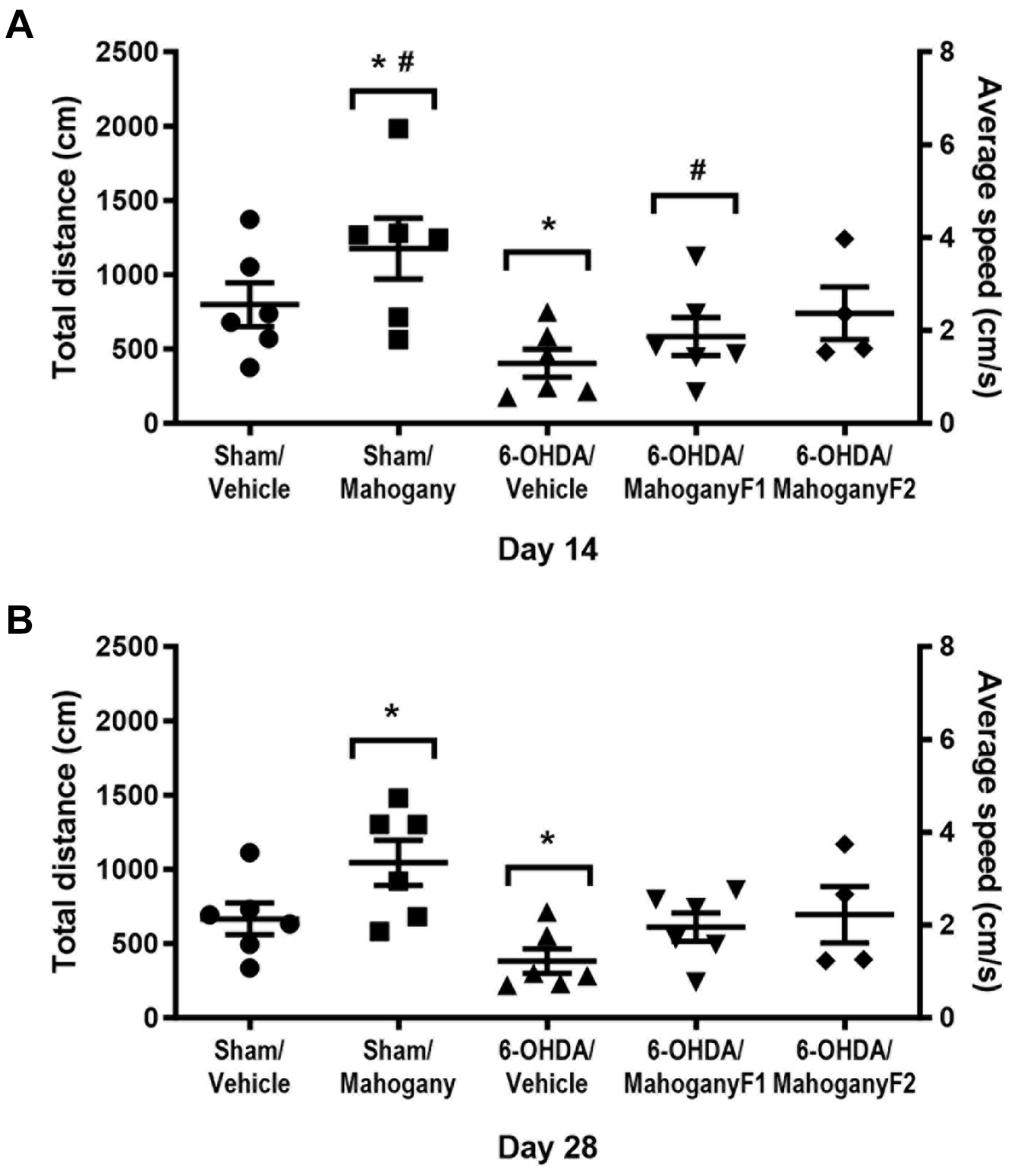
Evaluation of exploratory activity in the open field test of animals injected with 6-OHDA or vehicle and treated with aqueous extract of the mahogany leaf. Total distance traveled (cm) and mean speed (cm/s). **(A)** Tests performed on the 14th post-surgery day. The symbols indicate: *sham/mahogany vs. 6-OHDA/vehicle, total distance traveled, *p* = 0.0078, and mean speed, *p* = 0.0109; sham/mahogany vs. 6-OHDA/mahogany F1, total distance traveled, *p* = 0.0491. **(B)** Tests performed on the 28th post-surgery day. The symbol indicates the following: *sham/mahogany vs. 6-OHDA/vehicle, total distance traveled, *p* = 0.0052, and mean speed, *p* = 0.0103. The data are expressed in mean ± SEM. One-way ANOVA followed by Tukey’s *post hoc* test, and statistical significance was defined with 95% confidence (*p* < 0.05).

The total distance traveled (cm) and average speed (cm/s) obtained in the open field test were used as parameters of innate exploratory activity. As shown in Figure 3A, on the 14th day, the 6-OHDA/mahogany F2 group had a displacement of 740.1 ± 176.8 cm and an average speed of 2.48 ± 0.58 cm/s. The 6-OHDA/mahogany F1 group had a displacement of 600.3 ± 142.4 cm and an average speed of 2.0 ± 0.47 cm/s, whereas the 6-OHDA/vehicle group had a displacement of only 403.8 ± 94.7 cm, with an average speed of 1.35 ± 0.32 cm/s. Although there was no significant difference between the groups injected with 6-OHDA, the 6-OHDA/mahogany F1 and 6-OHDA/mahogany F2 groups showed a tendency toward motor recovery. Furthermore, the horizontal displacement was aligned with the motor performance of the sham/vehicle group (distance traveled, 798.2 ± 146.4 cm, and average speed, 2.70 ± 0.48 cm/s). The sham/mahogany group showed a mild stimulating effect on ambulation, whereas the 6-OHDA/vehicle group showed horizontal hypoactivity; these two factors significantly accentuated the differences between the groups (distance traveled *p* = 0.0078; average speed, *p* = 0.0109).

In the test carried out on the 28th day post-surgery, the 6-OHDA/mahogany F2 group maintained the trend of motor recovery, with a distance covered of 694.5 ± 189.7 cm and an average speed of 2.33 ± 0.63 cm/s and 6-OHDA/mahogany F1, with a distance covered of 611.2 ± 95.2 cm and an average speed of 2.05 ± 0.32 cm/s; while the 6-OHDA/vehicle group continued to be hypoactive, presenting a displacement of 382.7 ± 82.5 cm and an average speed of 1.27 ± 0.28 cm/s. There was no significant difference between the groups injected with 6-OHDA. The sham/mahogany group showed a mild stimulating effect on ambulation, whereas the 6-OHDA/vehicle group showed horizontal hypoactivity; these two factors significantly accentuated the differences between the groups (distance traveled, *p* = 0.0052; average speed, *p* = 0.0103).

However, it was observed that the motor performance of the 6-OHDA/mahogany F2 and 6-OHDA/mahogany F1 groups remained equivalent to that of the sham/vehicle group (distance traveled, 666.1 ± 107.0 cm and average speed, 2.20 ± 0.36 cm/s) (Figure 3B).

**Figure 4:**
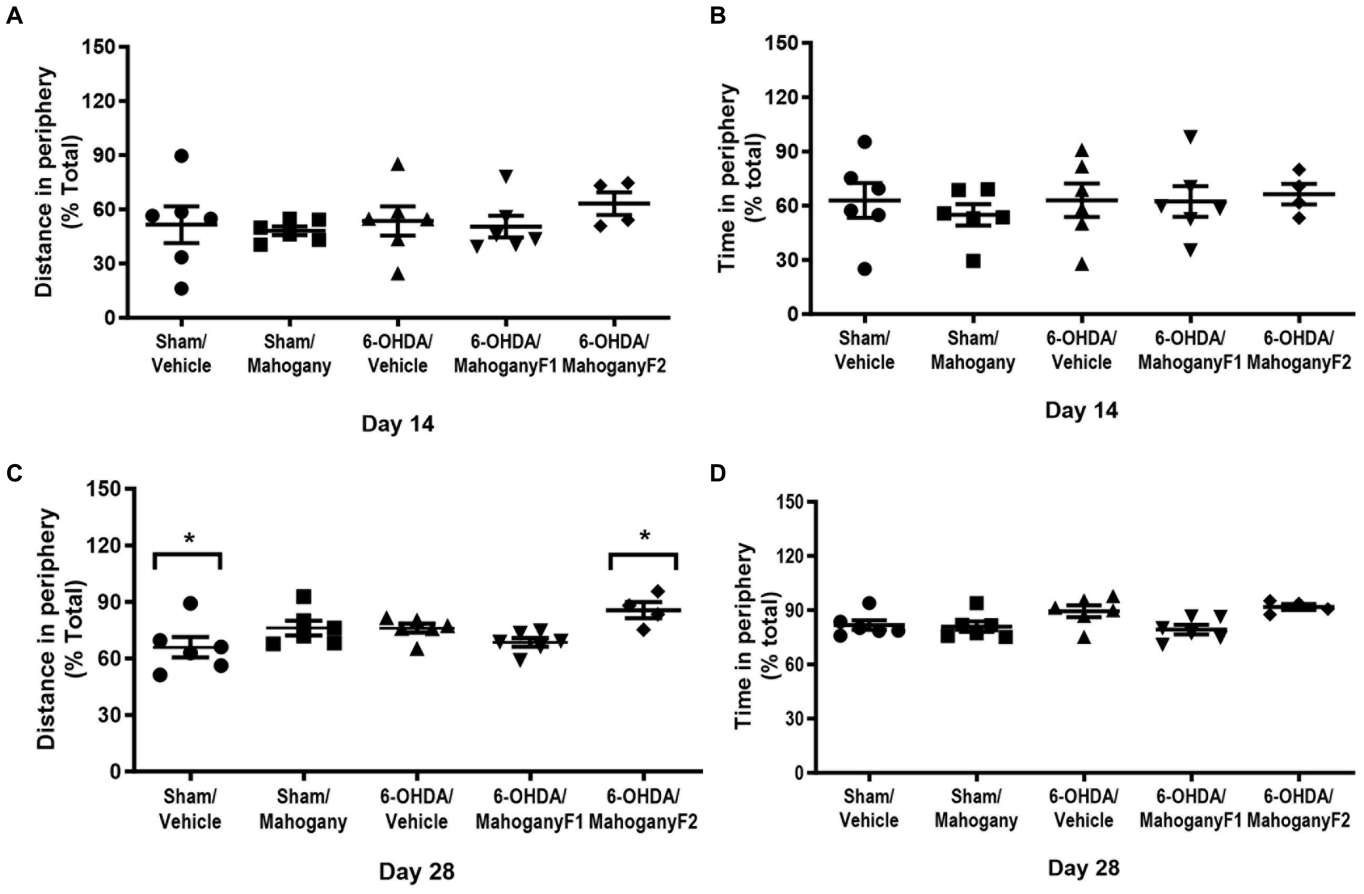
Evaluation of anxious-like behavior in the open field test of animals injected with 6-OHDA or vehicle and treated with aqueous extract of the mahogany leaf or vehicle. **(A)** Distance traveled in the periphery on day 14; **(B)** time spent in the periphery on day 14; **(C)** distance traveled in the periphery on day 28; **(D)** time spent in the periphery on day 28. The asterisks above the bars indicate significant differences between the groups. *sham/vehicle vs. 6-OHDA/mahogany, *p* = 0.0191. The data are expressed in mean ± SEM. One-way ANOVA followed by Tukey’s *post hoc* test was used, and statistical significance was defined with 95% confidence (*p* < 0.05).

The percentages of displacement and time on the periphery of the arena on the 14th day post-surgery were as follows: sham/vehicle, 51.6 ± 10.2 and 62.9 ± 9.6%; sham/mahogany F1, 48.2 ± 2.4% and 54.9 ± 5.9%; 6-OHDA/vehicle, 53.6 ± 8.1% and 63.0 ± 5.8%; sham/mahogany F1, 50.5 ± 6.0 and 62.3 ± 8.5; and 6-OHDA/mahogany F2, 63.2 ± 6.2 and 66.4 ± 5.8%, respectively. There was no significant difference between the groups; therefore, the unilateral injection of 6-OHDA into the dorsal striatum did not reproduce aspects related to anxiety-like behavior in the open field test (Figures 4A,B).

On the 28th day, the 6-OHDA/mahogany F2 group showed a more pronounced horizontal displacement in the periphery (85.6 ± 4.3%) and was significantly different from the sham/vehicle group, which had a displacement in the periphery of 65.9 ± 5.4% (*p* = 0.0191). Regarding the percentage of time spent in the periphery, there was no significant difference between the groups (Figures 4C,D).

#### Assessment of exploratory activity and anxiety-like behavior in the elevated plus maze (EPM)

**Figure 5:**
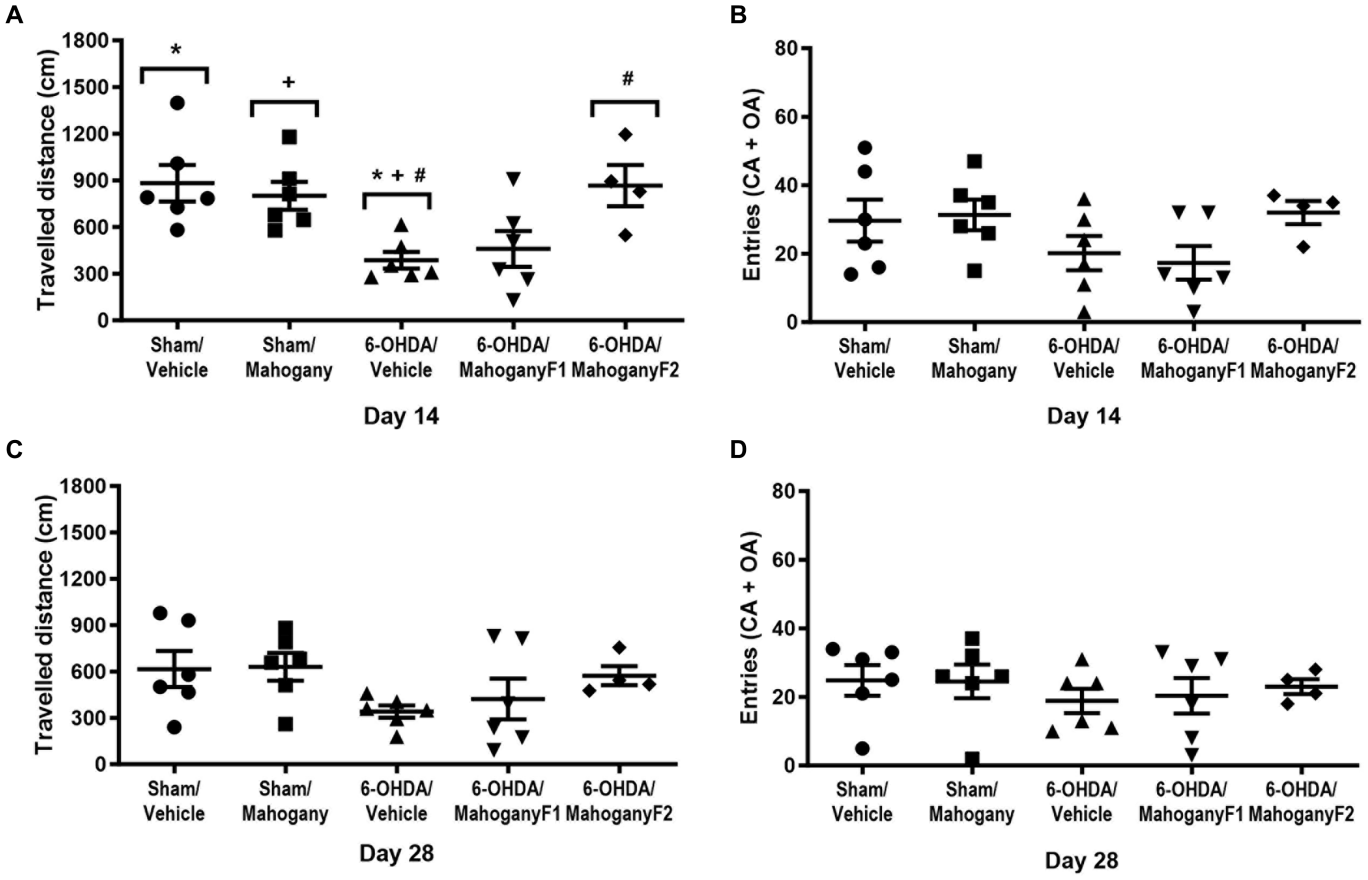
Evaluation of exploratory behavior in the Elevated plus maze test, on days 14 and 28 after surgery, of animals injected with 6-OHDA or vehicle and treated with aqueous extract of the mahogany leaf or vehicle. **(A)** Total distance traveled (CM), 14th day. **(B)** Frequency of entries in the open and closed arms, 14th day. **(C)** Total distance traveled (cm), 14th day. **(D)** Frequency of entries in the open and closed arms on day 14. The symbols above the bars indicate significant differences between the groups. * sham/vehicle vs. 6-OHDA/vehicle, *p* = 0.0125; ^+^ sham/mahogany vs. 6-OHDA/vehicle, *p* = 0.0286; ^#^6-OHDA/vehicle vs. 6-OHDA mahogany F2, *p* = 0.0221. The data are expressed in mean ± SEM. One-way ANOVA followed by Tukey’s *post hoc* test was used, and statistical significance was defined with 95% confidence (*p* < 0.05).

Exploratory activity was assessed through the total distance traveled and the number of entries into the open and closed arms (EOCA). As shown in Figure 5A, on the 14th day, in the 6-OHDA/mahogany F2 group, the total distance traveled was 866.4 ± 123.9 cm, which represented a significant improvement compared with the 6-OHDA/vehicle group, which had a displacement of 386.6 ± 54.0 cm (*p* < 0.05). The motor performance in the 6-OHDA/mahogany F2 group was compatible with the results presented by the sham/vehicle (881.2 ± 117.5 cm) and sham/mahogany (792.3 ± 94.4 cm) groups.

Regarding the number of entries in open and closed arms, the groups presented the following averages: sham/vehicle, 29.7 ± 6.2; sham/mahogany, 31.1 ± 4.5; 6-OHDA/vehicle, 20.2 ± 5.0; 6-OHDA/mahogany F1, 18.7 ± 4.5; and 6-OHDA/mahogany F2, 31.3 ± 3.1. There was no significant difference when comparing the groups. However, we observed that the 6-OHDA/mahogany F2 group had better motor performance than the model group, although the difference was not significant (Figure 5B).

With the repetition of the test on the 28th day, a reduction in general activity was identified in all groups. Thus, the displacement in the 6-OHDA/mahogany F2 group was 573.0 ± 62.1 cm, 6-OHDA/mahogany F1 was 442.0 ± 132.4 and 6-OHDA/vehicle was 340.7 ± 39.8 cm. Although there was no significant difference between the groups, our data showed that even with the repetition of the test, 6-OHDA/mahogany F2 maintained a strong trend of motor improvement in relation to the model group (Figure 5C).

Regarding the entries in the open and closed frequency parameter, the averages were as follows: sham/vehicle, 24.8 ± 4.5; sham/mahogany, 24.5 ± 4.9; 6-OHDA/vehicle, 18.8 ± 3.5; 6-OHDA/mahogany F1, 20.3 ± 5.2; and 6-OHDA/mahogany F2, 23.2 ± 2.2. There was no statistically significant difference when comparing the groups (Figure 5D). We emphasize that the decrease in the apparatus exploration rate may have been influenced by habituation with repeated tests.

In the two elevated plus maze assessments, the parameters of time in closed arms (CA) and time in open arms (OA) were not affected in the groups injected with 6-OHDA in relation to the sham groups. Likewise, the frequency of visits in the open and closed arms was not affected.

Taken together, these results show that emotionality indices were not influenced by 6-OHDA infusion. These elevated plus maze results corroborate the data obtained in the open field, in which we observed an absence of anxiety-related behavior.

### Histopathological analysis

#### Optical density of TH+ terminals in the dorsal and ventral striatum

**Figure 6:**
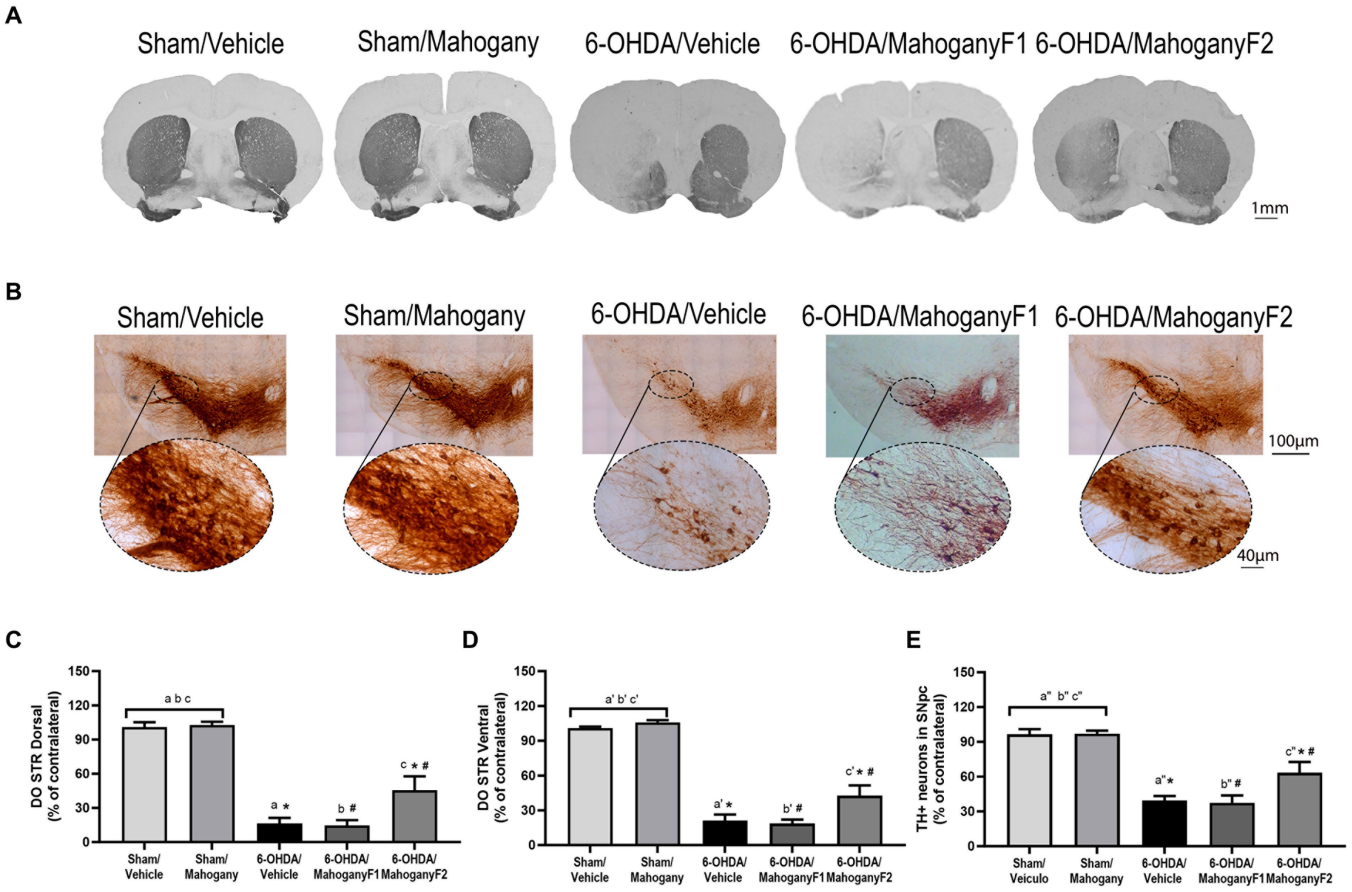
Histopathological analysis of animals injected with 6-OHDA or vehicle and treated with aqueous extract of the mahogany leaf or vehicle. **(A)** Photomicrographs of coronal sections of the brain representative of each group. **(B)** Photomicrographs of coronal sections of the ipsilateral ventral midbrain and SNpc. **(C)** Optical density analysis of TH+ fibers in the dorsal striatum. The symbols indicate: ^a^sham/vehicle *vs* 6-OHDA/Vehicle, *p* < 0,0001; ^b^sham/Vehicle *vs* 6-OHDA/mahogany F1, *p* < 0,0001; ^c^sham/Vehicle *vs* 6-OHDA/mahogany F2, *p* < 0,0001; ^a^sham/mahogany *vs* 6-OHDA/Vehicle, *p* < 0,0001; ^b^sham/mahogany *vs* 6-OHDA/mahogany F1, *p* < 0,0001; ^c^sham/mahogany *vs* 6-OHDA/mahogany F2, *p* < 0,0001; ^*^6-OHDA/mahogany F2 *vs* 6-OHDA/vehicle, *p* = 0,0074; ^#^6-OHDA/mahogany F2 *vs* 6-OHDA/mahogany F1, *p* = 0,0074. **(D)** Analysis by optical density of TH+ fibers in ventral striatum. The letters above the bars indicate: ^a’^sham/Vehicle *vs* 6-OHDA/Vehicle, *p* < 0,0001; ^b’^sham/Vehicle *vs* 6-OHDA/mahogany F1, *p* < 0,0001; ^c’^sham/Vehicle *vs* 6-OHDA/mahogany F2, *p* < 0,0001; ^a’^sham/mahogany *vs* 6-OHDA/Vehicle, *p* < 0,0001; ^b’^sham/mahogany *vs* 6-OHDA/mahogany F1, *p* < 0,0001; ^c’^sham/mahogany *vs* 6-OHDA/mahogany F2, *p* < 0,0001; ^*^6-OHDA/mahogany F2 *vs* 6-OHDA/Vehicle, *p* = 0,0291; ^#^ 6-OHDA/mahogany F2 *vs* 6-OHDA/mahogany F1, *p* = 0,0098. **(E)** Estimate of the percentage of TH^+^ neurons in the SNpc. The symbols indicate: ^a”^sham/Vehicle *vs* 6-OHDA/Vehicle, *p* < 0,0001; ^b”^sham/Vehicle *vs* 6-OHDA/mahogany F1, *p* < 0,0001; ^c”^sham/Vehicle *vs* 6-OHDA/mahogany F2, *p* = 0,0033; ^a”^sham/mahogany *vs* 6-OHDA/Vehicle, *p* < 0,0001; ^b”^sham/mahogany *vs* 6-OHDA/mahogany F1, *p* < 0,0001; ^c”^sham/Vehicle *vs* 6-OHDA/mahogany F2, *p* = 0,0029; ^*^6-OHDA/mahogany F2 *vs* 6-OHDA/Vehicle, *p* = 0,0482; ^#^6-OHDA/mahogany F2 *vs* 6-OHDA/mahogany F2, *p* = 0,0273. The data are expressed in mean ± SEM. One-way ANOVA followed by Tukey’s *post hoc* test was used, and statistical significance was defined with 95% confidence (*p* < 0.05).

For each group, we recorded photomicrographs of representative coronal sections of the striatum. As expected, animals injected with ascorbic acid did not show loss of TH+ immunoreactivity, and a higher density of TH+ fibers was observed in the 6-OHDA/mahogany F2 group than in the 6-OHDA/vehicle and 6-OHDA/mahogany F1 groups (Figure 6A).

The 6-OHDA/vehicle group showed a severe and significant reduction in TH^+^ expression in the injected side and only 16.3 ± 5.0% immunostaining in the dorsal striatum. A similar histopathological aspect was identified in the 6-OHDA/mahogany F1 group, with 17.7 ± 4.5% TH+ fibers. In the 6-OHDA/mahogany F2 group, the expression of TH+ in the terminals represented 45.6 ± 12.2% of the remaining TH-immunolabeled fibers (6-OHDA/vehicle vs. 6-OHDA/mahogany F2, *p* < 0.008) (Figure 6C).

Analysis of the optical density of TH+ fibers in the ventral striatum showed that in the 6-OHDA/vehicle group, the percentage of TH^+^ immunostaining remained significantly lower than that in the other groups, with only 21.0 ± 5.5%. A similar histopathological aspect was identified in the 6-OHDA/mahogany F1 group, with 19.7 ± 3.4% of TH+ fibers. In the 6-OHDA/mahogany F2 group, immunostaining was 42.6 ± 8.9%, with an additional contribution of 21.6 ± 6.8% of TH+ fibers in relation to the model (6-OHDA/vehicle vs. 6-OHDA/mahogany F2, *p* = 0.0291) (Figure 6D).

Taken together, the optical density results of the dorsal and ventral striatum confirm that the lesion was more severe in the 6-OHDA/mahogany F1 group than in the 6-OHDA/mahogany F2 group. In addition, the aqueous extract of the mahogany leaf treatment did not interfere with the course of the lesion.

#### Estimation of the number of TH+ neurons in the substantia nigra pars compacta (SNpc)

In each group, we recorded photomicrographs of the left ventral mesencephalon. As expected, the sham groups did not show loss of TH^+^ immunoreactivity, and the largest volume of TH+ neuron perikarya and branches in the SNpc was observed in the 6-OHDA/mahogany F2 group compared with the 6-OHDA/vehicle group (Figure 6B).

The percentage of TH+ neurons in the SNpc in the 6-OHDA/vehicle group was 35.5 ± 3.9%; a similar histopathological aspect was identified in the 6-OHDA/mahogany F1 group, with 37.4 ± 6.4%, whereas the percentage of remaining dopaminergic neurons in the 6-OHDA/mahogany F2 group was 64.1 ± 8.9% (6-OHDA/vehicle vs. 6-OHDA/mahogany F2, *p* = 0.0051). These data confirm that in the 6-OHDA/mahogany F1 group, lesions were more severe, whereas those in the 6-OHDA/mahogany F2 group were milder.

In the 6-OHDA/mahogany F2 group, neurodegeneration was partial, and compared with the sham/vehicle group, the percentage deficit of TH^+^ neurons in the SNpc was 32.5 ± 6.8% (*p* = 0.0008), and compared with the sham/mahogany group, it was 32.9 ± 6.8% (*p* = 0.0007) (Figure 6E).

#### Estimation of the number of microglia IBA-1+ in the dorsal and ventral striatum

**Figure 7:**
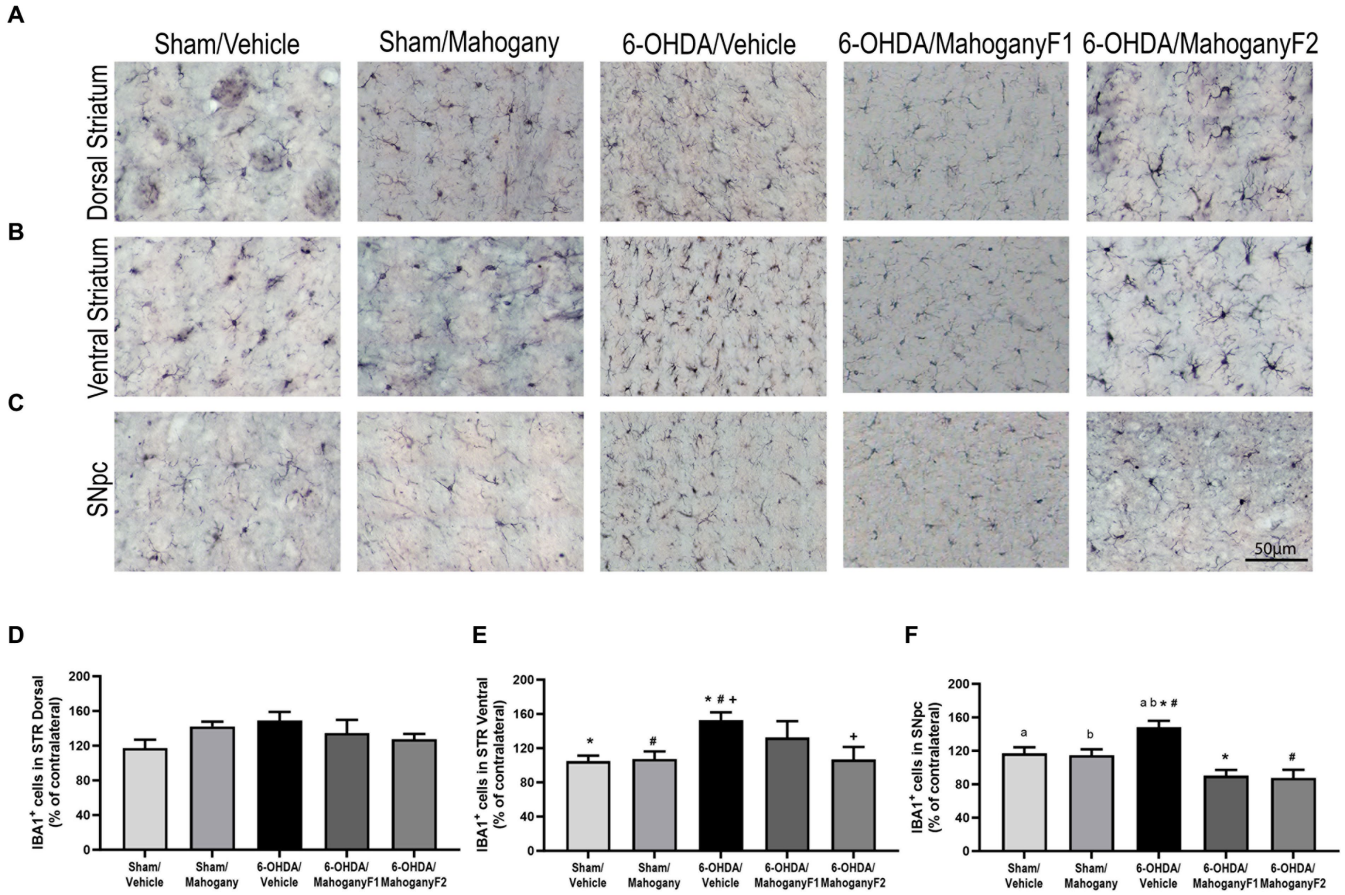
Estimation of the percentage of IBA-1+ microglia in the striatum of animals injected with 6-OHDA or vehicle and treated with aqueous extract of the mahogany leaf or vehicle. **(A)** Photomicrographs of coronal sections of the ipsilateral dorsal striatum representative of each group. **(B)** Photomicrographs of coronal sections of the ipsilateral ventral striatum representative of each group. **(C)** Photomicrographs of coronal sections of the ipsilateral SNpc, representative of each group. **(D)** Percentage of IBA-1+ cells in the dorsal striatum. There was no statistically significant difference between the groups. **(E)** Percentage of IBA-1+ cells in the ventral striatum. Symbols indicate: ^+^6-OHDA/vehicle vs. 6-OHDA/mahogany F2, *p* = 0.0214; *6-OHDA/vehicle vs. sham/vehicle, *p* = 0.0069; ^#^6-OHDA/vehicle vs. sham/mahogany, *p* = 0.0107. **(F)** Percentage of IBA-1+ cells in SNpc. Symbols indicate: ^#^6-OHDA/vehicle vs. 6-OHDA/mahogany F2, *p* = 0.0003; ^a^6-OHDA/vehicle vs. sham/vehicle, *p* = 0.0487; ^b^6-OHDA/vehicle vs. sham/mahogany, *p* = 0.0303. Data are presented as mean ± SEM. One-way ANOVA followed by Tukey’s *post hoc* test and statistical significance was defined at a 95% confidence level (*p* < 0.05).

From each group, we recorded photomicrographs of the left dorsal striatum (Figure 7A), left ventral striatum (Figure 7B), and left SNpc (Figure 7C). The percentages of IBA-1+ cells in the dorsal striatum were as follows: sham/vehicle, 117.2 ± 9.6%; sham/mahogany, 142.5 ± 5.5%; 6-OHDA/vehicle, 148.9 ± 9.9%; 6-OHDA/mahogany F1, 134.6 ± 15.1%; and 6-OHDA/mahogany F2, 127.6 ± 5.9%. There was no significant difference when comparing the groups (Figure 7D). Despite the lack of statistical significance, the results show that there was an increase in the population of microglia in relation to the contralateral side in the five groups of this study.

These data indicate that in the dorsal striatum, the mechanical injury caused by the needle path contributed to the exacerbated microglial response, whereas the antioxidant potency of the aqueous extract of the mahogany leaf could not overcome the severe local neuroinflammation triggered acutely by the infusion of 6-OHDA.

The percentage of IBA-1^+^ cells in the ventral striatum of the 6-OHDA/mahogany F2 group was 106.8 ± 12.7%, which was significantly lower than that of the 6-OHDA/vehicle group, with 152.7 ± 4.3% (*p* = 0.0214). This result shows that there was a 45.9 ± 14.2% reduction in neuroinflammation in the ventral striatum in the 6-OHDA/mahogany F2 group compared with the model group, suggesting that aqueous extract of the mahogany leaf treatment reduced the progression of neuroinflammation to the ventral striatum (Figure 7E). While the percentage of IBA-1^+^ cells in the ventral striatum of the 6-OHDA/mahogany F1 group was 132 ± 18.9%, there was no significant difference compared with the model group.

#### Estimation of the number of microglia in the substantia nigra pars compacta (SNpc)

The percentage of IBA-1+ cells in the SNpc in the 6-OHDA/mahogany F2 group was 87.6 ± 9.6%, which was significantly lower than that in the 6-OHDA/vehicle group, which 148.0 ± 7.9% (*p* = 0.003). Thus, these data indicate that treatment with aqueous extract of the mahogany leaf attenuated microglial proliferation by 60.4 ± 12.0% in the 6-OHDA/mahogany F2 group compared with the model group. While the percentage of IBA-1+ cells in the SNpc in the 6-OHDA/mahogany F1 group was 90.1 ± 7.0%, it was also significantly lower than that in the 6-OHDA/vehicle group (*p* = 0.001). These data indicate attenuated microglial proliferation by 57.8 ± 10.5% in the 6-OHDA/mahogany F1 group.

Treatment with the aqueous extract of the mahogany leaf consistently reduced the progression of neuroinflammation in the dopaminergic nigrostriatal pathway of animals injected with 6-OHDA. On the other hand, ascorbic acid injection did not influence microglial proliferation in the SNpc in the sham/vehicle group, whose estimation of IBA-1+ cells was 117.5 ± 7.6%, and in the sham/mahogany group, it was 114.7 ± 7.1% (Figure 7F).

#### Densitometry of GFAP+ expression in the dorsal and ventral striatum

**Figure 8:**
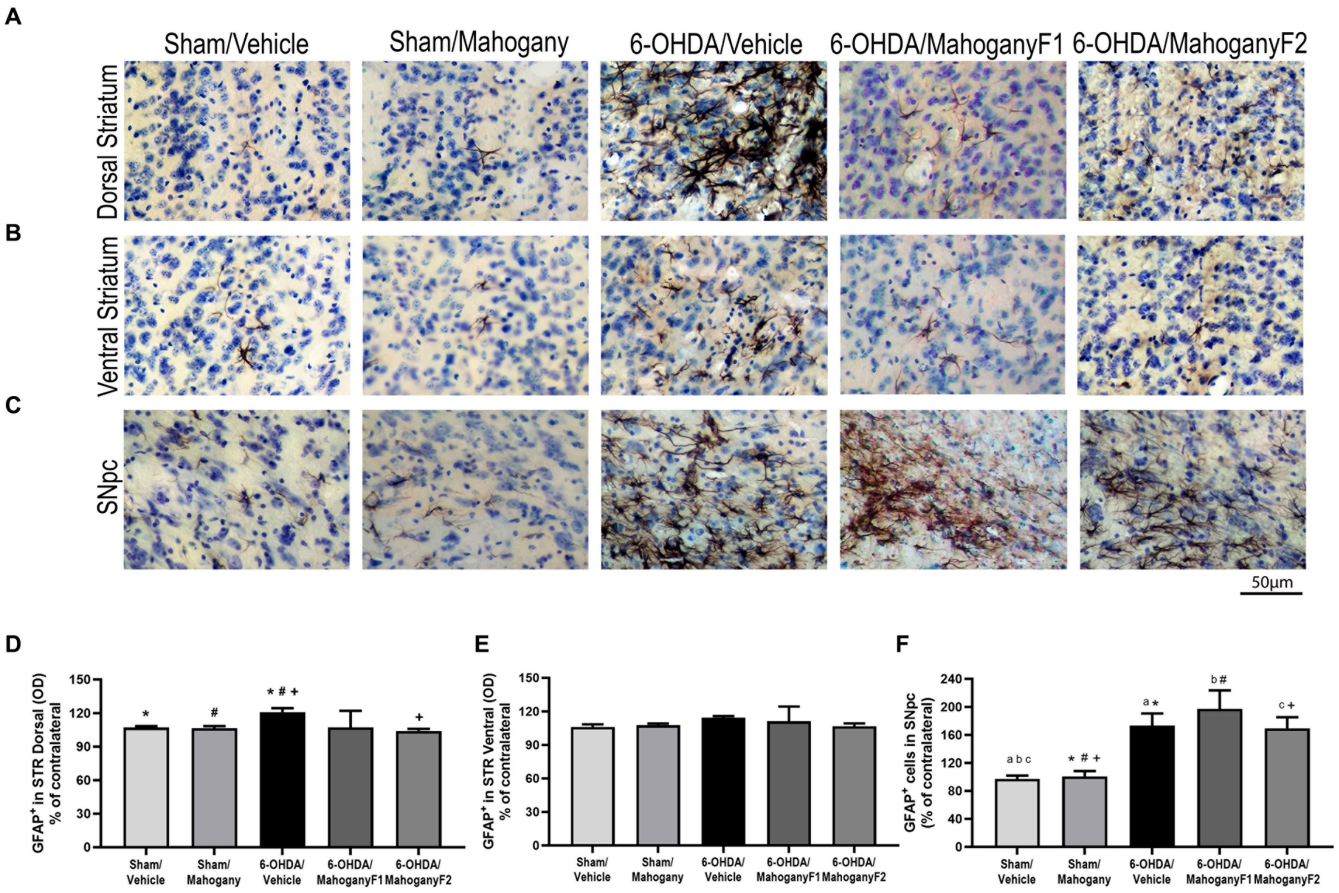
Analysis by optical density of GFAP+ immunostaining in the dorsal and ventral striatum and estimate of the percentage of GFAP+ cells in the SNpc of animals injected with 6-OHDA or vehicle and treated with aqueous extract of the mahogany leaf or vehicle. **(A)** Photomicrographs of coronal sections of the ipsilateral dorsal striatum, representative of each group. **(B)** Photomicrographs of coronal sections of the ipsilateral ventral striatum, representative of each group. **(C)** Photomicrographs of coronal sections of the ipsilateral SNpc, representative of each group. **(D)** Analysis by optical density of GFAP+ reactivity in the dorsal striatum. Symbols indicate: *sham/vehicle vs. 6-OHDA/vehicle, *p* = 0.0422; ^#^sham/mahogany vs. 6-OHDA/vehicle, *p* = 0.0422; ^+^6-OHDA/vehicle vs. 6-OHDA/mahogany F2, *p* = 0.0110. **(E)** Analysis by optical density of GFAP+ reactivity in the ventral STR. There was no statistically significant difference between the groups. **(F)** Estimation of the percentage of GFAP+ astrocytes in SNpc. Symbols indicate: ^a^sham/vehicle vs. 6-OHDA/vehicle, *p* = 0.0184; ^b^sham/vehicle vs. 6-OHDA/mahogany F1, *p* = 0.0184; ^c^sham/vehicle vs. 6-OHDA/mahogany F2, *p* = 0.0263; *sham/mahogany vs. 6-OHDA/vehicle, *p* = 0.0356; ^#^sham/mahogany vs. 6-OHDA/mahogany F1, *p* = 0.0042; ^+^sham/mahogany vs. 6-OHDA/mahogany F1, *p* = 0.0496. Data are presented as mean ± SEM. One-way ANOVA followed by Tukey’s *post hoc* test and statistical significance was defined at a 95% confidence level (*p* < 0.05).

From each group, we recorded photomicrographs of the left dorsal striatum (Figure 8A), left ventral striatum (Figure 8B), and left SNpc (Figure 8C). Optical density analysis in the dorsal striatum showed that GFAP^+^ cells in the 6-OHDA/mahogany F1group was 107.2 ± 14.9%, whereas in the 6-OHDA/mahogany F2 group, GFAP+ expression was 104.0 ± 1.8%, which was significantly lower than that in the 6-OHDA/vehicle group, which exhibited 120.9 ± 3.5% (*p* = 0.0110) (Figure 8D). Furthermore, the percentage of optical density in the dorsal striatum in the 6-OHDA/mahogany F2 group was statistically equivalent to that in the sham/vehicle group, with 105.9 ± 2.8%, and sham/mahogany, with 106.2 ± 3.6%. In addition, the 6-OHDA/vehicle group exhibited changes in astrocyte morphology, such as increased soma and thickness of primary processes (Figure 8A).

Regarding immunoreactivity for GFAP+ in the ventral striatum, the data show: sham/vehicle, 106.1 ± 2.3%; sham/mahogany, 107.6 ± 1.4%; 6-OHDA/vehicle, 114.3 ± 1.5%; 6-OHDA/mahogany F1, 111.1 ± 13.3%; and 6-OHDA/mahogany F2, 106.8 ± 5.1%. There was no significant difference between the groups. We observed that the astroglial response was restricted to the 6-OHDA infusion site and did not significantly extend to the ventral striatum (Figure 8E).

#### Estimation of the number of astrocytes in the substantia nigra pars compacta (SNpc)

GFAP+ cells in the SNpc in the groups injected with the toxin were as follows: 6-OHDA/vehicle, 173.3 ± 17.3%; 6-OHDA/mahogany F1, 197.3 ± 26.4%; and 6-OHDA/mahogany F2, 168.4 ± 16.5%. There was no significant difference between the groups injected with 6-OHDA.

However, the astroglial response in the SNpc of the 6-OHDA-injected groups remained significantly more pronounced compared to the sham/vehicle group, 97.3 ± 4.6% and sham/mahogany group, 100.7 ± 7.8%. The differences between the groups were as follows: 6-OHDA/vehicle vs. sham/vehicle, *p* = 0.0184; 6-OHDA/vehicle vs. sham/mahogany, *p* = 0.0356; 6-OHDA/mahogany F1 vs. sham/vehicle, *p* = 0.0184; 6-OHDA/mahogany F1 vs. sham/mahogany, *p* = 0.0042; 6-OHDA/mahogany F2 vs. sham/vehicle, *p* = 0.0263; and 6-OHDA/mahogany F2 vs. sham/mahogany, *p* = 0.0496 (Figure 8F). These data indicate that in this study, treatment with aqueous extract of the mahogany leaf did not modulate the expression of GFAP in the SNpc of animals injected with 6-OHDA.

Furthermore, we observed that in the groups injected with 6-OHDA, the astrocytes presented with morphological changes characteristic of reactive astrogliosis. These observations are in agreement with data provided in the literature, which show that 6-OHDA injection causes strong and robust astroglial activation, accompanied by permanent cellular changes.

## Discussion

Pamplona et al. (2015) conducted phytochemical and biological prospecting studies in the aqueous extract of mahogany leaf (AEML) and found 9 types of phenolic acids and 18 types of flavonoids with antioxidant and anti-inflammatory properties. In the present study, we evaluated the neuroprotective potential of this mahogany extract in a murine model of PD induced by 6-OHDA.

Unilateral injection of 6-OHDA into the striatum causes dopaminergic imbalance between the cerebral hemispheres, and the advancement of nigrostriatal neurodegeneration impacts the hypersensitivity of its receptors in the ipsilateral striatum (Meredith and Kang, 2006; Brooks and Dunnett, 2009). Therefore, motor pathways can be asymmetrically activated through the action of a dopaminergic agonist, generating rotational behavior toward the side contralateral to the lesion (Björklund and Dunnett, 2019; Buhidma et al., 2020). This rotational behavior in lesioned animals is highly reproducible and has been used to establish an index of motor recovery and neuroprotection after exposure to therapeutic drugs (Simola et al., 2007; Buhidma et al., 2020).

In our study, in the 6-OHDA/vehicle group, contralateral rotations became more frequent and vigorous with the progression of the lesion, confirming the successful injection and toxic effect of 6-OHDA in the dorsal striatum. The 6-OHDA/mahogany F1 group showed a significant increase in the average rotations in the second test with apomorphine, indicating a lesion comparable with the 6-OHDA/vehicle group, which was confirmed subsequently in our histopathological evaluation. Thus, EAML treatment did not protect against the progression of the nigrostriatal lesion. In contrast, the 6-OHDA/mahogany F2 group did not show any contralateral rotations after apomorphine challenge, which was compatible with a low degree of lesion in the dopaminergic nigrostriatal pathway, which was also confirmed by histopathology. These results in the F2 group could be due to a technical problem that interfered with the final lesion size after 6-OHDA injection, a neuroprotective effect of the EAML treatment, or a combination of both. At this point, we cannot rule out any of these alternatives.

Motor performance in PD unilateral animal models can also be evaluated using open field (Seibenhener and Wooten, 2015; Willard et al., 2015) and elevated plus maze tests (Carobrez and Bertoglio, 2005; La-Vu et al., 2020). In our study, the sham/mahogany group showed increased motor behavior compared with the 6-OHDA/vehicle group. Results from the elevated plus maze tests showed that the 6-OHDA/mahogany F2 group had significantly better motor performance than the model group in the first test on the 14th post-surgery day. The percentage of entries in the open arms was not affected in either group. In the second test, despite habituation, the 6-OHDA/mahogany F2 group maintained better motor performance than the 6-OHDA/vehicle group. These results are consistent with the data obtained in the open field test, which indicated better motor capacity, in agreement with a lower degree of degeneration in the nigrostriatal dopaminergic pathway in the F2 group compared with the 6-OHDA/vehicle and 6-OHDA/mahogany F1 groups.

In addition to motor disorders, anxiety is a non-motor symptom that affects approximately 35–40% of patients with PD and has a significant impact on patient quality of life (Kano et al., 2011; Dissanayaka et al., 2014; Khatri et al., 2020). Although non-motor symptoms have been little studied, especially in models of unilateral injury, the retrograde neurodegeneration induced by the infusion of 6-OHDA in the striatum resembles the initial stages of PD and has been used as a tool for investigating neuropsychiatric changes, such as anxiety (Breit et al., 2007). However, studies using different protocols regarding dosis, time, and site of injection have achieved divergent results in relation to anxiety-related behavior (Branchi et al., 2008; Tadaiesky et al., 2008; Prasad and Hung, 2020). In our study, there was no difference between the groups in anxiety-related parameters, both in the open field (not shown) and in the elevated plus maze tests. These results suggest that the observed differences in motor performance between the groups were not due to anxiety-like behavior.

In the histopathological analysis, we found significantly more TH^+^ fibers in the striatum and a higher percentage of dopaminergic neurons in the SNpc in the 6-OHDA/mahogany F2 group than in the 6-OHDA/vehicle and 6-OHDA/mahogany F1 groups. Therefore, the histopathological findings confirm the lower degree of lesion in the 6-OHDA/mahogany F2 group, which is compatible with the number of rotations in the apomorphine test, and improved motor performance in the open field and elevated plus maze tests compared with the 6-OHDA/vehicle and 6-OHDA/mahogany F1 groups.

In the present study, we found a significantly higher percentage of microglia in the dorsal striatum, ventral striatum, and SNpc in the 6-OHDA/vehicle group, indicating exacerbated neuroinflammation induced by the toxin. However, in the dorsal striatum, increased microglia staining was also observed in the control groups that were not injected with the toxin, and we attribute this effect to the mechanical injury caused by the needle used for the infusion of the toxin or vehicle. The 6-OHDA groups treated with AEML showed consistently decreased microglial proliferation in the ventral striatum and SNpc, indicating a significant impact on controlling neuroinflammation independent of lesion severity. The absence of apomorphine-induced rotations in the F2 group, even with a loss of 36.7% of nigral dopaminergic neurons, may be due to a decrease in neuroinflammation after treatment with the mahogany extract.

Another important signature of the 6-OHDA-induced injury model is astrocyte hyperreactivity during nigrostriatal dopaminergic projection (Mathiisen et al., 2010; Gradisnik and Velnar, 2023). Astrocytes respond to different types and intensities of perturbations in the CNS and can adopt various context-dependent phenotypes; however, changes in their molecular profile can be reversible (Escartin et al., 2021). *In vivo* studies that used the model of unilateral injection of 6-OHDA into the medial forebrain bundle to induce dopaminergic desensitization in the nigrostriatal pathway showed that in the ipsilateral SNpc, there was a significant increase in GFAP immunoreactivity, including an increase in cell volume and the number of astrocytes of the inflammatory phenotype (Kuter et al., 2018; Jiang et al., 2021).

In our study, the histopathological data from the 6-OHDA/Vehicle group are in agreement with the literature, as the areas in the dorsal striatum and SNpc occupied by hyper-reactive GFAP+ cells were significantly larger than those in the sham groups. Furthermore, these areas were marked by hypertrophic astrocytes soma, thicker primary processes, and densely overlapping branches. These notable morphological changes in astrocytes from the 6-OHDA/vehicle group were correlated with severe degeneration of the nigrostriatal dopaminergic pathway. In contrast, the 6-OHDA/Mahogany F2 group showed less hypertrophy of the soma and densely overlapping branches in the dorsal region, suggesting a modulating effect of AEML treatment. The exacerbation of GFAP+ immunoreactivity in the SNpc of the 6-OHDA/Mahogany F1 and 6-OHDA/Mahogany F2 groups seems to proceed from a distinct source of neuroinflammation because IBA-1+ immunoreactivity was significantly reduced in this nucleus. GFAP hyperreactivity, which diffusely occurred in the SNpc, could be a direct response of astrocytes to 6-OHDA, which reached the cell body via degenerating dopaminergic neurons, or a response to the neurodegenerative process itself.

The percentage of polyphenols in the aqueous extract of the mahogany leaf is significantly higher than that in leaf extracts from other Amazonian plants obtained by the same method (Silva et al., 2007). Some polyphenols found in the phytochemical constitution of mahogany (*Swietenia macrophylla*) are also part of the phytochemical constitution of other plants, and their antioxidant capacity has already been tested in other studies using models of nigrostriatal dopaminergic degeneration induced by toxins, such as: naringerin (Zbarsky et al., 2005; Lou et al., 2014; Kim et al., 2016); resveratrol (Jin et al., 2008; Khan et al., 2010; Wang et al., 2011); quercetin (Haleagrahara et al., 2011); rutin (Moshahid Khan et al., 2012); and myricetin (Ma et al., 2007). In this study, it is conceivable that several bioactive compounds in the aqueous extract of mahogany leaf may jointly contribute to the observed beneficial effects. Further studies are necessary to better characterize their applicability for treating chronic degenerative diseases with inflammatory and oxidative bases, such as Parkinson’s disease.

## Data availability statement

The raw data supporting the conclusions of this article will be made available by the authors upon request without reservation.

## Ethics statement

The animal study was approved by Comissão de Ética no Uso de Animais, Federal University of Pará. The study was conducted in accordance with the local legislation and institutional requirements.

## Author contributions

VC: Formal analysis, Investigation, Methodology, Writing – original draft, Writing – review & editing, Conceptualization, Data curation, Supervision. AV-A: Investigation, Methodology, Writing – original draft, Supervision, Writing – review & editing. RM: Investigation, Writing – review & editing, Methodology, Supervision. CM: Investigation, Writing – original draft, Methodology. NM: Investigation, Writing – original draft, Methodology. MS: Validation, Writing – review & editing, Conceptualization, Supervision. JP: Validation, Writing – review & editing, Conceptualization, Supervision. GB: Supervision, Validation, Writing – review & editing, Conceptualization, Methodology. JF: Writing – review & editing, Methodology, Validation. EY: Conceptualization, Formal analysis, Methodology, Supervision, Writing – review & editing, Validation.
